# Therapeutic importance of sulfated polysaccharides from seaweeds: updating the recent findings

**DOI:** 10.1007/s13205-012-0061-9

**Published:** 2012-04-15

**Authors:** Seema Patel

**Affiliations:** Department of Biotechnology, Lovely Professional University, Jalandhar, 144402 Punjab India

**Keywords:** Sulfated polysaccharides, Antioxidant, Antitumor, Anticoagulant, Antiviral

## Abstract

Seaweeds, being prolific sources of bioactive components have garnered unprecedented interest in recent times. The complex polysaccharides from the brown, red and green seaweeds possess broad spectrum therapeutic properties. Especially, the sulfated polysaccharides, viz*.* fucans, carrageenans and ulvans have exhibited strong antioxidant, antitumor, immunostimulatory, anti-inflammatory, pulmonary fibrosis anticoagulant/antithrombotic, lipid lowering, antiviral, antibacterial, antiprotozoan, hyperplasia prevention, gastrointestinal, regenerative and nano medicine applications. Considering the immense biomedical prospects of sulfated polysaccharides, the profound and emerging functional properties published in recent times will be discussed here with experimental evidences. The limitations of the seaweed-derived sulfated polysaccharides in healthcare will be summarized. Strategies to maximize extraction and bioavailability will be pondered.

## Introduction

**Fig. 1:**
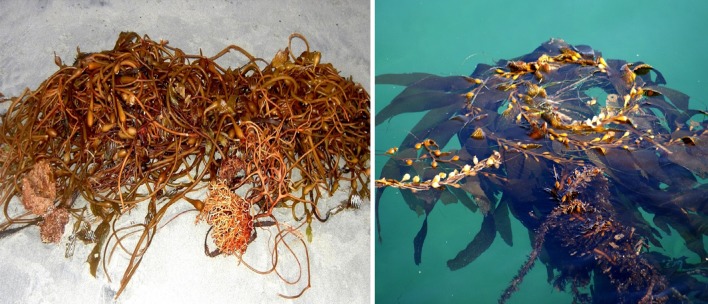
Seaweeds growing on the California Coast of the Pacific Ocean

**Fig. 2:**
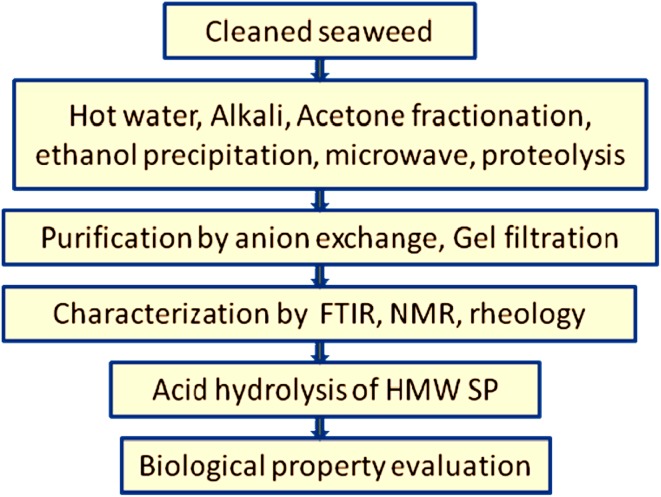
A flowchart depicting the sequential steps for sulfated polysaccharide preparation and biological activity evaluation

In recent years, much attention has been focused on polysaccharides isolated from natural sources. During the last decade, numerous bioactive polysaccharides with interesting functional properties have been discovered from seaweeds (Fig. 1). Several algal species belonging to phaeophyta, rhodophyta and chlorophyta divisions have been recognized as crucial sources of sulfated polysaccharides (SP). These SP constitute an important ingredient of cell walls and get harvested by suitable extraction or precipitation method, followed by purification, characterization and biological studies (Fig. 2). The biological features of the SP reported till now are antioxidant, antitumor, immunomodulatory, inflammation, anticoagulant, antiviral, antiprotozoan, antibacterial, antilipemic. Currently, the regenerative medicine and tissue engineering application of the SP has become a hot research area. Jiménez-Escrig et al. (2011) have reviewed the vital role of SP from seaweeds in human health.

**Fig. 3:**
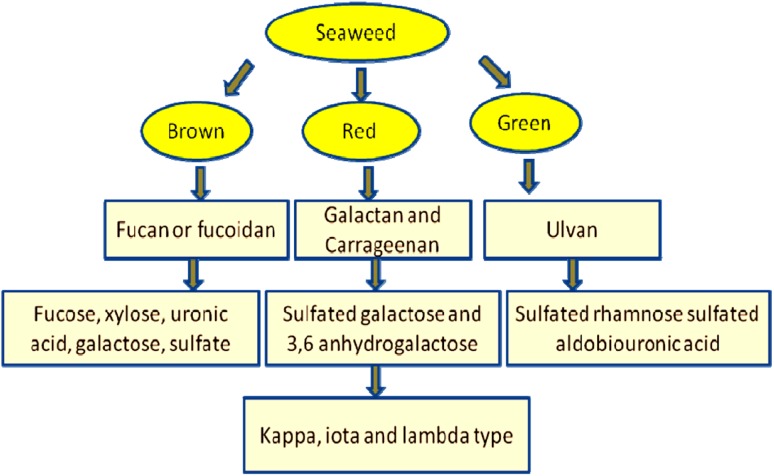
Classification of the bioactive sulfated polysaccharides

Bioactive SP extracted from seaweeds can be classified into three types. The major fucan yielding brown seaweeds genera are *Fucus*, *Sargassum*, *Laminaria*, *Undaria*, *Lessonia*, *Dictyota, Dictyopteris,Ascophyllum, Eclonia, Canistrocarpus, Lobophota, Turbinaria, Padina, Adenocystis*, *Sphacelaria*, *Cystoseira*, etc*.* Fucan represents a family of water soluble, SP rich in sulfated l-fucose, extracted from extracellular matrix of these weeds (Li et al. 2008; Costa et al. 2011a). Fucoidan, the sulfated alpha-l-fucan (term often interchangeably used with fucans) has demonstrated a wide range of pharmacological activities. Carrageenans are a family of linear SP, extracted from red seaweeds, viz. *Gracialaria, Gigartina, Gelidium, Lomentaria, Corallina, Champia, Solieria, Gyrodinium, Nemalion,Sphaerococcus, Boergeseniella, Sebdenia, Scinaia*, etc*.* This group of polysaccharides has a backbone of alternating 3-linked β-d-galactose and 4-linked α-d-galactose residues (Tuvikene et al. 2006). Three categories of carrageenans, kappa (κ), iota (ι), and lambda (λ) have been identified till now based on their sulfation degree, solubility and gelling properties (Leibbrandt et al. 2010). Ulvan is the major water soluble, sulfated polysaccharide, extracted from the cell wall of green algae, viz*.Ulva*, *Enteromorpha*, *Monostroma*, *Caulerpa*, *Codium*, *Gayralia*. Ulvans are composed of disaccharide repetition moieties made up of sulfated rhamnose linked to either glucuronic acid, iduronic acid, or xylose and represent about 8–29 % of the algal dry weight (Lahaye and Robic 2007). The above-described SP have been illustrated in Fig. 3.

The therapeutic mechanisms of these SP vary, hence it is yet to be studied precisely. For anticoagulation potency, the formation of the SP/protease protein complex and the associated non-specific polar interaction between the negatively and positively charged groups in the polysaccharide and protein is responsible for anticoagulant activity. The anticoagulant activity is mainly attributed to thrombin inhibition mediated by heparin cofactor II, with different effectiveness depending on the compound. Similarly, selectin blockade, inhibition of enzyme and complement cascade seem to be the triggers leading to anti-inflammation. Combating viral infection has been shown by adsorption and internalization steps (Kim et al. 2011, 2012).

Ion exchange, gel filtration, FTIR, NMR analyses are employed to elucidate the composition and structure of SP. Cutting edge technologies, viz*.* MTT assay, flow cytometry, western blot analysis, BCA protein assay, SDS-PAGE and gelatin zymography has been employed for analysis of their functional properties (Jiang and Guan 2009). Although the use of the seaweed-derived polysaccharides in food industry as thickening, gelling agents, and stable excipients for control release tablets are well established, the clinical use is still to gain ground. Manifold increase in the published findings on this aspect in recent time is evidence enough for the craze over this highly promising domain. Recently, Senni et al. (2011) have reviewed the advancement in therapeutic potential of marine polysaccharides. However, this report was not confined to seaweeds and dealt only with the tissue engineering applications. Also, Wijesekara et al. (2011) have published an overview of clinically crucial SP extracted from marine algae. Keeping with the hot trend and in an attempt to present a new perspective, the present review summarizes the up-to-date literature data and discusses the pharmaceutical potential of different SP extracted from brown, red and green seaweeds.

## Therapeutic potential of sulfated polysaccharides

Researchers across the globe are waking up to the discovery that seaweed-derived bioactive products are a storehouse of healthy attributes. Recent times have seen a surge in interest to tap these unexploited marine sources to develop novel therapeutics. The SP of algal origin have exhibited miraculous biological properties. The common seaweeds, their SP and observed bioactivity spectra have been presented in Table 1.

**Table 1:** The studied seaweeds, their bioactive sulfated polysaccharides and therapeutic properties

Biological properties	Seaweed	Sulfated polysaccharide	References
Antioxidant	*Gracilaria birdiae* (red)	Fucoidan	Souza et al. (2012)
	*Fucus vesiculosus* (brown)	Galactan	Veena et al. (2007)
	*Gigartina skottsbergii* (red)	Carrageenan	Barahona et al. (2011)
	*Schizymenia binderi* (red)	Rhamnan	Magalhaes et al. (2011)
	*Lessonia vadosa* (brown)		Costa et al. (2011a, b)
	*Dictyopteris delicatula* (brown)		Wang et al. (2008)
	*Sargassum filipendula* (brown)		Devaki et al. (2009)
	*Laminaria japonica* (brown)		Camara et al. (2011)
	*Ulva lactuca* (green)		Hu et al. (2010)
	*Canistrocarpus cervicornis* (brown)		Yang et al. (2011)
	*Undaria pinnitafida* (brown)		Costa et al. (2010)
	*Corallina officinalis* (red)		
	*Corallinasertularioide* (red)		
	*Dictyotacervicornis* (brown)		
	*Sargassumfilipendula* (brown)		
	*Dictyopterisdelicatula* (brown)		
Antitumor	*Saccharina japonica* (brown)	Galactofucan	Vishchuk et al. (2011)
	*Undaria pinnatifida* (brown)	Mannoglucuronofucan	Costa et al. (2010)
	*Sargassumfilipendula* (brown)		Charles et al. (2007)
	*Dictyopterisdelicatula* (brown)		Ye et al. (2008)
	*Caulerpaprolifera* (green)		Croci et al. (2011)
	*Dictyotamenstrualis* (brown)		Ermakova et al. (2011)
	*Monostroma nitidum* (green)		Costa et al. (2011a, b)
	*Sargassum pallidum* (brown)		Magalhaes et al. (2011)
	*Laminaria saccharina* (brown)		Jin et al. (2010)
	*Ecklonia cava* (brown)		Lins et al. (2009)
	*Sargassum hornery* (brown)		Foley et al. (2011)
	*Costaria costata* (brown)		Haneji et al. (2005)
	*Sargassum filipendula* (brown)		
	*Dictyopteris delicatula* (brown)		
	*Champia feldmannii* (red)		
	*Ascophyllum nodosum* (brown)		
	Cladosiphon *okamuranus* Tokida		
Immunostimulatory	*Enteromorpha prolifera* (green)	Fucoidan	Kim et al. (2011, 2012)
	*Champia feldmannii* (red)	κ-carrageenan	Lins et al. (2009)
	*Fucus vesiculosus* (brown)	Oligosaccharides	Kawashima et al. (2011)
	*Kappaphycus striatum* (red)		Kima and Joo (2008)
Antiinflammation and antinociceptive	*Solieria filiformis* (red)	Galactan	de Araújo et al. (2011)
	*Gelidium crinale* (red)	Mannoglucuronofucans	Farias et al. (2011)
	*Sargassum hemiphyllum* (brown)	κ-carrageenan	de Sousa et al. (2011a)
	*Gracilaria cornea* (red)	Oligosaccharides	Hwang et al. (2011)
	*Gracilaria birdiae* (red)		Coura et al. (2011)
	*Laminaria saccharina* (brown)		Croci et al. (2011)
	*Lobophora variegate* (brown)		Medeiros et al. (2008)
	*Turbinaria ornata* (brown)		Ananthi et al. (2009)
	*Padina gymnospora* (brown)		Marques et al. (2012)
			Jiang and Guan (2009)
Anticoagulation and antithrombosis)	*Ecklonia cava* (brown)	Arabinogalactans	Wijesinghe et al. (2011)
	*Dictyota cervicornis* (brown)	Rhamnan	Costa et al. (2010)
	*Caulerpa cupresoides* (green)	Galactan	Ciancia et al. (2007)
	*Codium fragile* (green)		Li et al. (2011)
	*Codium vermilara* (green)		Mao et al. (2008)
	*Monostroma latissimum* (green)		Camara et al. (2011)
	*Monostroma nitidum* (green)		Albuquerque et al. (2004)
	*Canistrocarpus cervicornis* (brown)		Pushpamali et al. (2008)
	*Dictyota menstrualis* (brown)		Croci et al. (2011)
	*Lomentaria catenata* (red)		
	*Laminaria saccharina* (brown)		
Lipid lowering	*Ulva lactuca* (green)	Fucoidan	Kim et al. (2010)
	Sargassum polycystum (brown)		Sathivel et al. (2008)
	Sargassum wightii (brown)		Raghavendran et al. (2005)
	*Laminaria japonica* (brown)		Huang et al. (2010)
Antiviral (Influenza, herpes, HIV)	*Gyrodinium impudium* (red)	Galactan	Ghosh et al. (2009)
	*Nemalion helminthoides* (red)	Mannans	Kim et al. (2011, 2012)
	*Gayralia oxysperma* (green)	Heterorhamnan	Recalde et al. (2009)
	*Sphaerococcus coronopifolius* (red)	Xylomannan sulfate	Cassolato et al. (2008)
	*Boergeseniella thuyoides* (red)	Xylogalactofucan	Bouhlal et al. (2011)
	*Sebdenia polydactyla* (red)	Xylomannan	Bandyopadhyay et al. (2011)
	*Sphacelaria indica* (brown)		Mandal et al. (2007)
	*Cystoseira indica* (brown)		
	*Grateloupia indica* (red)		Chattopadhyay et al. (2007)
	*Laminaria angustata* (brown)		Trinchero et al. (2009)
	*Adenocystis utricularis* (brown)		Mandal et al. (2008)
	*Scinaia hatei* (red)		
Antibacterial (ampicillin resistant *E. coli)* Antiprotozoan (cryptosporidiosis, malaria)	*Kappaphycus alvarezii* (red)	Fucoidan	Kumaran et al. (2010)
	*Padina boergessenii* (brown)		Maruyama et al. (2007)
	*Undaria pinnatifida* (brown)		Chen et al. (2009)
Prevent hyperplasia	Brown seaweeds	Fucoidan	Hlawaty et al. (2011)
			Freguin-Bouilland et al. (2007)
Cause gastrointestinal contraction	*Halymenia floresia* (red)	Galactan	Graça et al. (2011)
	Cladosiphon okamuranus Tokida (brown)	Fucoidan	Matsumoto et al. (2004)
Regenerative and nano medicine	Brown seaweeds	Fucoidan	Sezer et al. (2008)
	*Ulva rigida* (green)	Ulvan	Murakami et al. (2010)
			Nakamura et al. (2008)
			Fukuta and Nakamura (2008)
			Toskas et al. 2011)

### Antioxidant

Souza et al. (2012) isolated a SP by aqueous extraction from the red seaweed *Gracilaria birdiae* and observed that the slimy substance exhibits moderate antioxidant properties as measured by DPPH free-radical scavenging effect. Veena et al. (2007) evaluated the efficacy of fucoidan from edible seaweed *Fucus vesiculosus* in Wistar rats (5 mg/kg body wt.). Advocation of the SP enhanced the antioxidant status, thereby preventing membrane injury and averting stone formation. Barahona et al. (2011) evaluated the antioxidant capacity of sulfated galactans from red seaweed *Gigartina skottsbergii* and *Schizymenia binderi*, commercial carrageenans, and fucoidan from brown seaweed *Lessonia vadosa* by the oxygen radical absorbance capacity (ORAC) method. Fucoidan from *L. vadosa* and the sulfated galactan from *S. binderi* exhibited the highest antioxidant capacity. The antioxidant capacity was also evaluated by ABTS and hydroxyl radical scavenging assays. *Corallinasertularioide*, *Dictyotacervicornis*, *Sargassumfilipendula* and *Dictyopterisdelicatula* were studied and found to have SP having immense antioxidant potential in the form of total antioxidant, reducing power and ferrous ion chelating activities (Costa et al. 2010). Two SP fractions rich in galactose and xylose from *Corallina officinalis* demonstrated considerable antioxidant properties (Yang et al. 2011). Hu et al. (2010) isolated two sulfated rhamnose-rich polysaccharide fractions from *Undaria pinnatifida* and evaluated their antioxidant abilities in vitro. It was revealed that the SP possessed strong antioxidant properties. Ye et al. (2008) evaluated the antioxidant activities of SP from *Sargassum pallidum* by DPPH (2,2-diphenyl-1-picrylhydrazyl)-free-radical scavenging assay and reported activity, though low at the tested concentration. Camara et al. (2011) extracted heterofucans from *Canistrocarpus cervicornis* by proteolytic digestion followed by sequential acetone precipitation. The SP exhibited total antioxidant capacity, low hydroxyl radical scavenging activity, good superoxide radical scavenging efficiency and excellent ferrous chelating ability. Devaki et al. (2009) studied the liver mitochondrial and microsomal fraction from rats to evaluate the antioxidative effect of oral gavaging with *Ulva lactuca* polysaccharide extract (200 mg/kg body weight, daily for 21 days). Electron microscopy of rat liver tissue intoxicated with d-galactosamine revealed the swelling and loss of mitochondrial cristae. However, the rats pre-treated with the SP overcame the d-galactosamine challenge without significant abnormality of TCA, microsomal enzymes and mitochondria structural aberrations. These results suggested that the SP play crucial role in stabilizing the functional status of mitochondrial and microsomal membrane by prevention of the oxidative stress induced by d-galactosamine. Fucoidan was extracted from *Laminaria japonica* through anion-exchange column chromatography and their antioxidant activities were investigated. Superoxide and hydroxyl radical scavenging activity, chelating ability and reducing power analysis showed that all fractions possessed considerable antioxidant activity (Wang et al. 2008). Gao et al. (2011) investigated the effects of fucoidan on improving learning and memory impairment in rats induced by infusion of beta-amyloid peptide, Aβ (1–40) and its possible mechanisms. The results indicated that fucoidan could ameliorate Aβ-induced cognitive disorders in neural maladies like Alzheimer’s. The mechanisms appeared to regulate the cholinergic system (increasing the activity of choline acetyl transferase), reduce the oxidative stress (reduced malondialdehyde content in hippocampal tissue of brain) and inhibit the cell apoptosis (increase of Bcl-2/Bax ratio and a decrease of caspase-3 activity). Hong et al. (2011) investigated the protective effect of fucoidan on dimethylnitrosamine-induced liver fibrogenesis in rats. When administered (100 mg/kg, 3 times per week), fucoidan improved liver fibrosis by inhibiting the expression of transforming growth factor beta 1 [TGF-β (1)]/Smad3 and the tissue inhibitor of metalloproteinase 1 (TIMP-1), and increasing the expression of metalloproteinase-9 (MMP-9). Fucoidan also significantly decreased the accumulation of the extracellular matrix and collagen, confirming its anti-fibrotic effect. Costa et al. (2011b) obtained five sulfated heterofucans from *S. filipendula* by proteolytic digestion followed by sequential acetone precipitation, which displayed considerable antioxidant potential. Magalhaes et al. (2011) obtained six families of SP from seaweed *D. delicatula* employing above-mentioned protocols, followed by molecular sieving on Sephadex G-100. Some fractions of the heterofucans showed high ferrous ion chelating activity and some fractions showed reasonable reducing power, about 53.2 % of the activity of vitamin C. These results clearly indicate the beneficial effects of SP from seaweeds in antioxidant status of consumers.

### Antitumor

Vishchuk et al. (2011) isolated fucoidans from brown seaweeds *Saccharina japonica* and *U. pinnatifida* and tested their antitumor activity against human breast cancer T-47D and melanoma SK-MEL-28 cell lines. The highly branched partially acetylated sulfated galactofucan, built up of (1 → 3)-α-l-fucose residues from *S. japonica* and *U. pinnatifida* distinctly inhibited proliferation and colony formation in both breast cancer and melanoma cell lines in a dose-dependent manner. These results indicated that the fucoidan from the studied seaweeds may be a potential approach toward cancer treatment. After 72-h incubation of HeLa cell with SP (0.01–2 mg/ml), the proliferation was inhibited between 33.0 and 67.5 % by *S. filipendula*; 31.4 and 65.7 % by *D.delicatula*; 36.3 and 58.4 % by *Caulerpaprolifera*, and 40.2 and 61.0 % by *Dictyotamenstrualis*. Costa et al. (2010) inferred that the antiproliferative efficacy of SP positively correlated with the sulfate content. In Sprague–Dawley rats fed with *Monostroma nitidum* diet, significant increase in UGT1A1 and UGT1A6 mRNA levels was found, indicating potential application in chemoprevention medicine (Charles et al. 2007). Ye et al. (2008) evaluated the antitumor activities of SP from *S. pallidum* by MTT [3-(4,5-dimethylthiazol-2-yl)-2,5-diphenyltetrazolium bromide] assay, which showed a significantly high antitumor activity against the human hepatocellular carcinoma (HepG2), human lung adenocarcinoma epithelial (A549) and human gastric carcinoma (MGC-803) cells. Croci et al. (2011) explored the possible antitumor activities of SP from the brown seaweed *Laminaria saccharina.* The incorporation of the parent SP and the sulfated fucans into Matrigel plugs containing melanoma cells induced a significant reduction in hemoglobin content as well as the frequency of tumor-associated blood vessels. Also, these two SP administrations resulted in a significant reduction of tumor growth when inoculated into mice. The sulfated fucan fraction markedly inhibited breast cancer cell adhesion to human platelet-coated surfaces. Ermakova et al. (2011) showed that fucoidans from brown algae *Eclonia cava*, *Sargassum hornery* and *Costaria costata* play an inhibitory role in colony formation in human melanoma and colon cancer cells. Costa et al. (2011b) observed antiproliferative activity of fucan from *S. filipendula* against HeLa cells by MTT test. The heterofucan was extracted from the brown seaweed by proteolytic digestion followed by sequential acetone precipitation. This SP showed antiproliferative activity on Hela cells and induced apoptosis by mitochondrial release of apoptosis-inducing factor (AIF) into cytosol. In addition, it decreased the expression of anti-apoptotic protein Bcl-2 and increased expression of apoptogenic protein Bax. Magalhaes et al. (2011) obtained six families of SP from seaweed *D. delicatula* by proteolytic digestion, followed by acetone fractionation and molecular sieving on Sephadex G-100. A fraction of the heterofucan showed high antiproliferative activity inhibiting almost 100 % of HeLa cell proliferation. Jin et al. (2010) investigated the effects of fucoidan on the apoptosis of human promyeloid leukemic cells and fucoidan-mediated signaling pathways. Fucoidan induced apoptosis of human promyelocytic leukemia (HL-60), human promyelocytic (NB4) and THP-1 (human acute monocytic leukemia) cell line. Fucoidan treatment of HL-60 cells induced activation of caspases 8, 9, and 3, the cleavage of Bid, and altered mitochondrial membrane permeability. Buthionine-[*R*,*S*]-sulfoximine rendered HL-60 cells more sensitive to fucoidan. It was concluded that the activation of MEKK1, MEK1, ERK1/2 and JNK, depletion of glutathione and production of NO are important mediators in fucoidan-induced apoptosis of human leukemic cells. Lins et al. (2009) investigated the in vitro and in vivo antitumor properties of a SP isolated from the seaweed *C. feldmannii*. The SP did not show any significant in vitro cytotoxicity at the experimental dose, but showed in vivo antitumor effect. The inhibition rates of sarcoma 180 tumor development were 48.62 and 48.16 % at the doses of 10 and 25 mg/kg, respectively. It also increased the response elicited by anti-cancer drug, 5-fluorouracil (5-FU) from 48.66 to 68.32 %. Though liver and kidney were moderately affected, the enzymatic activity of alanine aminotransferase or urea/creatinine levels was not disturbed. Leucopenia associated with 5-fluorouracil treatment was prevented when the chemotherapeutic was administered along with SP. An unfractionated fucoidan was extracted from the brown alga *Ascophyllum nodosum* and its effect on the apoptosis of human HCT116 colon carcinoma cells was studied and the signaling pathways involved were investigated. Fucoidan decreased cell viability and induced apoptosis of the carcinoma cells, through activation of caspases 9 and 3 and the cleavage of PARP (Foley et al. 2011). Haneji et al. (2005) examined the effect of fucoidan from the brown seaweed *Cladosiphon okamuranus* Tokida against an incurable form of cancer, the adult T-cell leukemia (ATL). It was observed that fucoidan inhibited the growth of peripheral blood mononuclear cells of ATL patients and caused apoptosis of HTLV-1-infected T-cell lines through a cascade of down regulations. In vivo treatment of the cancer transplanted in mice also showed partial inhibition of the tumors. Now that, cancer has assumed an epidemic proportion and the treatment scenario is still bleak, the SP from the marine weeds hold the promise for novel anticancer formulae.

### Immunostimulatory

Water-soluble SP extracted from *Enteromorpha prolifera* and fractionated using ion-exchange chromatography was investigated to determine their in vitro and in vivo immunomodulatory activities. Some fractions stimulated a macrophage cell line Raw 264.7 inducing considerable nitric oxide (NO) and various cytokine production via up-regulated mRNA expression. The in vivo experiment results showed increase in IFN-γ and IL-2 secretions, suggesting that the SP is a strong immunostimulator. It is implied that the SP can activate T cells by up-regulating Th-1 response (Kim et al. 2011). Lins et al. (2009) demonstrated that SP extracted from *C. feldmannii* is an immunomodulatory agent, evident from the increase in the production of specific antibodies. Kawashima et al. (2011) demonstrated that fucoidan enhances the probiotic effects of lactic acid bacteria on immune functions. In vitro test results showed that fucoidan amplified interferon (IFN)-γ production mediated by IL-12 production from Peyer’s patch and spleen cells in response to a strain of LAB, *Tetragenococcus halophilus* KK221. In vivo study showed that Th1/Th2 immunobalance was significantly improved by oral administration of both fucoidan and KK221 to ovalbumin-immunized mice. Kima and Joo (2008) observed that fucoidan from *F. vesiculosus* shows immunostimulating and maturing effects on dendritic cells (DCs) via a pathway involving nuclear factor-κB (NF-κB). κ-Carrageenan oligosaccharides from red algae *Kappaphycus striatum* have immunomodulation effects on S180 tumor-bearing mice. The sulfated derivative (200 μg/g/day) showed an increase in natural killer cells (NK cells) up to 76.1 %. It suggested that chemical modification (especially sulfation) of carrageenan oligosaccharides can enhance their antitumor effect and boost their antitumor immunity. Yuan et al. (2011) reported not only the capacity of SP to elicit cellular immunity but also the importance of chemical modification of the parent polysaccharide.

### Anti-inflammation/antinociception/inhibition of pulmonary fibrosis

de Araújo et al. (2011) studied the antiinflammatory and antinociception (less sensitivity to painful stimulus) properties of seaweed *Solieria filiformis* in vivo. Male Swiss mice pre-treated with the SP, on receiving an injection of 0.8 % acetic acid, 1 % formalin or 30 min prior to a thermal stimulus, showed significantly reduced number of writhes. It showed antinociceptive action through a peripheral mechanism; however, did not show any significant anti-inflammatory effect. The SP from the brown seaweed *Spatoglossum schroederi* was assayed for the antinociceptive effect on Swiss mice. The SP purified by anion-exchange chromatography inhibited both phases of the formalin test. In the first phase the maximum 45 % reduction in paw licking was observed. This inhibitory effect suggested a mixed mechanism similar to morphine, which was not confirmed in the hot-plate test. It was concluded that the pronounced antinociceptive effect of SP could be developed as a new source of analgesic drugs (Farias et al. 2011). The SP galactan extracted from the red marine alga *Gelidium crinale* was purified by ion-exchange chromatography and tested by intravenous route in rodent experimental models of inflammation and nociception. The anti-inflammatory activity was evaluated in the model of rat paw edema induced by different inflammatory stimuli. Antinociceptive effect was assessed in models of nociception/hyperalgesia elicited by chemical (formalin test), thermal (hot plate), and mechanical (von Frey) stimuli in mice. It was observed that SP inhibited the time course of dextran-induced paw edema and showed a maximal effect at 1 mg/kg (42 %). At the highest dose, the SP also inhibited the paw edema induced by histamine (49 %) and phospholipase A(2) (44 %). The galactan inhibited both neurogenic and inflammatory phases of the formalin test and the treatment was well tolerated by the test animals (de Sousa et al. 2011a). Hwang et al. (2011) explored SP from brown seaweed *Sargassum hemiphyllum* for possible anti-inflammatory effect. The SP was administered against the mouse macrophage cell line (RAW 264.7) activated by lipopolysaccharide (LPS). The secretion profiles of pro-inflammatory cytokines, including IL-1β, IL-6, TNF-α, and NO, were found significantly to be reduced in 1–5 mg/ml dose ranges of SP treatments. RT-PCR analysis suggested that the SP inhibits the LPS-triggered mRNA expressions of IL-β, iNOS and COX-2 in a dose-dependent manner. It was concluded that the anti-inflammatory properties of SP may be attributed to the down-regulation of NF-κB in nucleus. Coura et al. (2011) evaluated the effects of SP from the red seaweed *Gracilaria cornea* in nociceptive and inflammatory mice models. At all tested doses, the SP significantly reduced nociceptive responses, as measured by the number of writhes. In a formalin test, the SP significantly reduced licking time in both phases of the test at a dose of 27 mg/kg. In a hot-plate test, the antinociceptive effect was observed only in animals treated with 27 mg/kg of SP, suggesting that the analgesic effect occurs through a central action mechanism at the highest dose. The lower doses of SP (3 and 9 mg/kg) caused only a slight reduction in neutrophil migration in the rat peritoneal cavity but significantly inhibited paw edema induced by carrageenan, especially at 3 h after treatment. Reduction in edema was confirmed by myeloperoxidase activity in the affected paw tissue. After 14 consecutive days of intraperitoneal administration of the SP (9 mg/kg), the biochemical, hematological and histopathological evaluations of the internal organs are performed and no systemic damage was found. de Sousa et al. (2011b) investigated the involvement of the hemoxygenase-1 (HO-1) pathway in the anti-inflammatory action of a SP from the red seaweed *G. birdiae.* The SP was administered at various concentrations to Wistar rats and observed that at 10 mg/kg concentration, it exerted an anti-inflammatory effect. A remarkable decrease in leukocytes in the peritoneal cavity was also observed. The SP also reduced the paw edema induced by carrageenan and inhibited the paw edema induced by dextran in the first half-hour. The *O*-sulfated mannoglucuronofucans and sulfated fucan fractions from the brown seaweed *L. saccharina* were evaluated for possible treatment of inflammation in vivo. Both types of SP exhibited inhibition of leukocyte rush into the sites of inflammation in the murine models (Croci et al. 2011). Medeiros et al. (2008) extracted a sulfated heterofucan from the brown seaweed *Lobophora variegata* by proteolytic digestion, followed by acetone fractionation, molecular sieving, and ion-exchange chromatography. The fucoidan revealed that it inhibits leukocyte migration to the inflammation site. Ear swelling caused by croton oil was also inhibited when sulfated polysaccharides from *F. vesiculosus* and *L. variegata* were used. Ananthi et al. (2009) investigated the anti-inflammatory effect of crude SP from brown alga *Turbinaria ornata* against carrageenan-induced paw edema in rats and vascular permeability in mice. Oral administration of SP reduced the paw edema and showed inhibitory effect on vascular permeability considerably, in a dose-dependent manner. SP extracted from brown algae *Padina gymnospora* showed efficacy in reducing leukocyte influx into the peritoneal cavity in mice at 10 mg/kg body weight, causing a decrease of 60 %, without any cytotoxicity (Marques et al. 2012). Idiopathic pulmonary fibrosis is a pathological condition characterized by accumulation of excess fibroblasts, deposition of collagen and inflammation in lungs. The pro-fibrogenic cytokine transforming growth factor-beta 1 (TGF-beta1) has attracted much attention for its potential role in the etiology of this serious lung injury. MS80, a new kind of sulfated oligosaccharide extracted from seaweed, inhibits TGF-beta1-induced pulmonary fibrosis in vitro and bleomycin-induced pulmonary fibrosis in vivo. The oligosaccharide competitively inhibited heparin/HS-TGF-beta1 interaction through its high binding affinity for TGF-beta1, also arrested human embryo pulmonary fibroblast (HEPF) cell proliferation and collagen deposition. MS80 proved to be a potent suppressor of bleomycin-induced rat pulmonary fibrosis in vivo (Jiang and Guan 2009). Du et al. (2010) reported that efficacy of MS80 lies in targeting the CD40 signal pathway by blocking RIP2. The precise mechanism of functionality is not clear; nevertheless, the sulfated polysaccharides studied above promise therapeutic potential in inflammatory disorders.

### Anticoagulation

Batteries of assays for assessment of anticoagulation properties of SP from seaweeds have been conducted in recent times. Tests ranging from activated partial thromboplastin time (APTT), thrombin time (TT), prothrombin time (PT), antithrombin to anticoagulation factor Xa activities have been performed and compared with heparin. Wijesinghe et al. (2011) purified a SP from brown seaweed *Ecklonia cava* and investigated its anticoagulant activity in vitro and in vivo. It extended the coagulation time in Wistar rats in a dose- and time-dependent manner. Costa et al. (2010) evaluated in vitro anticoagulant activities of marine algae SP by APTT test. *D. cervicornis* SP prolonged the coagulation time, only 1.4-fold lesser than Clexane^®^, a low molecular weight commercial heparin. In the prothrombin time (PT) test, which evaluates the extrinsic coagulation pathway, *Caulerpa cupresoides* showed aggression. *Codium fragile* and *Codium vermilara* water-soluble sulfated arabinogalactans prevented coagulation, but they induced platelet aggregation. It was observed that anticoagulant activity was higher in SP samples with higher sulfate content. In this regard, *C. vermilara* proved to be superior with a higher degree of sulfation and arabinose content (Ciancia et al. 2007). The hot water extract of green alga *Monostroma latissimum* gives a sulfated rhamnan polysaccharide with an anticoagulant activity. The anticoagulant activity as evaluated by assays of the APTT and thrombin time promises that it can be a potential source of anticoagulant (Li et al. 2011). Mao et al. (2008) isolated two sulfated, rhamnose-containing polysaccharides from marine green algae *M. nitidum* and evaluated their anticoagulant activities. The results showed that both the SP possess high anticoagulant activities, and were potent thrombin inhibitors mediated by heparin cofactor II. They also hastened thrombin and coagulation factor Xa inhibition by potentiating antithrombin III. Camara et al. (2011) extracted sulfated heterofucans from *C. cervicornis* which prolonged APTT. Four sulfated polysaccharides doubled APTT with only 0.1 mg/ml of plasma, only 1.25-fold less than Clexane^®^. Albuquerque et al. (2004) extracted heterofucans from the brown seaweed *D. menstrualis* by proteolytic digestion, followed by sequential acetone precipitation. The anticoagulant activities of these heterofucans were determined by APTT test. A fucan fraction (20 g/ml) demonstrated significant anticoagulant activity, about 4.88-fold lesser than Clexane^®^ (4.1 g/ml). Pushpamali et al. (2008) isolated a highly sulfated (21.76 %), 100–500 kDa molecular weight galactan anticoagulant from microbial-fermented freeze-dried red algae *Lomentaria catenata*. It demonstrated that the anticoagulant compound showed better efficacy than heparin and prolonged activity toward APTT and PT assays. Croci et al. (2011) studied that the SP from the brown seaweed *L. saccharina* shows promising activity on thrombosis. Fernández et al. (2012) studied the anticoagulation efficacy of sulfated β-d-mannan extracted from green seaweed *C. vermilara* and reported that higher sulfate content leads to more pronounced effect. Fucoidan has been proposed as a potential substitute of the anticoagulant heparin, with added merits. Unlike mammalian mucosa-derived heparin, fucoidan is extracted from plants, so less likely to contain infectious agents, such as viruses or prions (Boisson-Vidal et al. 1995)*.* The current findings promise a host of possible candidates for natural anticoagulant preparation.

### Lipid lowering

Fucoidan has been reported to affect the development of adipocytes. To elucidate the role of fucoidan in adipogenesis, its inhibitory effect on adipocyte differentiation via mitogen-activated protein kinase (MAPK) signaling pathway in 3T3-L1 preadipocytes was studied. Fucoidan treatment inhibited the adipocyte differentiation, evidenced by decreased lipid accumulation and down-regulation of adipocyte markers. Also, it inhibited the expression of adipogenic transcription factors, α (C/EBPα), γ (PPARγ) and AP2, crucial for adipocyte development (Kim et al. 2010). Sathivel et al. (2008) evaluated the anti-peroxidative and anti-hyperlipidemic property of *U. lactuca* polysaccharide extract against d-galactosamine (500 mg/kg body weight)-induced anomaly in rat. d-Galactosamine-intoxicated rats showed significant liver damage with acute aberration in serum lipid profile, hepatic protein thiols, deposits of lipid droplets and abnormal appearance of mitochondria. Rats pretreated with ulvan (30 mg/kg body weight/day/for 21 days) showed a significant inhibition against abnormality induced by d-galactosamine. The effect of *Sargassum polycystum* crude SP extract on lipid metabolism was examined against acetaminophen-induced hyperlipidemia in experimental rats. The prior oral administration of *S. polycystum* (200 mg/kg body wt./day for a period of 15 days) crude SP extract showed considerable prevention in the severe disturbances of lipid profile and metabolizing enzymes (serum lecithin cholesterol acyl transferase and hepatic triglyceride lipase) triggered by acetaminophen. Liver histology also supported their protective nature against fatty changes induced during acetaminophen intoxication (Raghavendran et al. 2005). Josephine et al. (2007) studied the possible capacity of SP in normalizing hyperlipidemia induced by the immunosuppressant drug cyclosporine A (25 mg/kg body weight, orally for 21 days) in Wistar rat kidney. As a side effect of the drug, lipid profile showed fluctuation resulting in nephrotoxicity manifested by the enhanced urinary excretion of urea, uric acid and creatinine. The SP-treated groups (5 mg/kg body weight, subcutaneously) showed a normalized lipid profile and lipid metabolizing enzymes. Moreover, this group of rats showed a normal concentration of urinary constituents. Huang et al. (2010) investigated the effect of fucoidan from *L. japonica* on hyperlipidemic rats. The SP reduced the concentration of serum total cholesterol, triglyceride and low-density lipoprotein cholesterol and increased the concentration of high-density lipoprotein cholesterol of the studied rats. The activities of lipoprotein lipase, hepatic lipoprotein and lecithin cholesterol acyltransferase were also enhanced. Above findings corroborate that the SP from seaweeds are ideal option for effective abatement of the lipid abnormalities.

### Antiviral

Many viruses display affinity for cell surface heparan sulfate proteoglycans playing crucial role in virus entry. This raises the possibility of the application of SP in antiviral therapy (Ghosh et al. 2009). Kim et al. (2012) purified a SP, p-KG03, from the red marine microalga, *Gyrodinium impudium*. The galactan conjugated to uronic acid and sulfated groups had showed inhibition of encephalomyocarditis virus. The inhibitory activity of the SP against influenza virus was examined. The results of a cytopathic effect reduction assay using MDCK cells demonstrated that p-KG03 exhibited the 50 % effective concentration (EC50) values of 0.19–0.48 μg/ml against influenza type A virus infection. The antiviral activity of p-KG03 was deduced to be directly associated with its interaction with viral particles, interfering with its adsorption and internalization into host cell. It was expected to be a candidate for antiviral drug development. The soluble fractions of a sulfated, (1 → 3)-linked α-d-mannans obtained by hot water extraction from *Nemalion helminthoides* showed appreciable antiherpetic activity (Recalde et al. 2009). A homogeneous branched sulfated heterorhamnan was obtained by aqueous extraction, followed by ultrafiltration from the green seaweed *Gayralia oxysperma* which exerted high specific activity against herpes simplex virus (HSV-1) (Cassolato et al. 2008). Treatment of human immunodeficiency virus type 1 (HIV-1), the dreaded etiological agent of AIDS poses tough challenges. The limitations encountered in therapeutic strategy are toxicity, resistance and high costs. Water-soluble sulfated galactans isolated from two red algae *Sphaerococcus coronopifolius* (Gigartinales, Sphaerococcaceae) and *Boergeseniella thuyoides* (Ceramiales, Rhodomelaceae) inhibited in vitro replication of the human immunodeficiency virus (HIV) at 12.5 μg/ml. In addition, the studied polysaccharides were capable of inhibiting the in vitro replication of HSV-1 on Vero cells. The adsorption step of HSV-1 to the host cell seemed to be the specific target for the SP action. While for HIV-1, these results suggest a direct inhibitory effect on HIV-1 replication by controlling the appearance of the new generations of virus and potential virucidal effect (Bouhlal et al. 2011). Ghosh et al. (2009) studied that xylomannan sulfate and its sulfated derivatives purified from *Sebdenia polydactyla* showed strong activity against HSV-1. The IC50 values were in the range 0.35–2.8 μg/ml and they did not exert cytotoxicity at concentrations up to 1,000 μg/ml. Many xylogalactofucan- and alginic acid-containing fractions from marine alga *Sphacelaria indica* showed antiherpetic activity. The IC50 values of their chemically sulfated derivatives against HSV-1 were in the range of 0.6–10 μg/ml and they lacked cytotoxicity at concentrations up to 200 μg/ml (Bandyopadhyay et al. 2011). Sulfated fucan-containing fractions isolated from the brown seaweed *Cystoseira indica* showed potent antiviral activity against HSV-1 and 2 HSV-2 without cytotoxicity for Vero cell cultures. Chemical, chromatographic and spectroscopic methods showed that the anti-herpetic activity of the SP is by inhibition of the virus adsorption (Mandal et al. 2007). Chattopadhyay et al. (2007) analyzed the SP fractions isolated from crude water extract of *Grateloupia indica* and showed their potent anti-HSV activity. The SP, xylogalactofucan fractions extracted from *Laminaria angustata*, after addition of sulfate groups showed enhanced capability to inhibit HSV-1. The IC50 values of these fractions against HSV-1 were in the range of 0.2–25 μg/ml and they lacked cytotoxicity at concentrations up to 1,000 μg/ml (Saha et al. 2012). SP fractions from brown seaweed *Adenocystis utricularis* were analyzed for their in vitro anti-HIV-1 activity. Two of the five studied fractions showed potent anti-HIV-1 activity both against wild type and drug-resistant HIV-1 strains, mediated by blockade of early events of viral replication (Trinchero et al. 2009). The antiviral activity was dependent on the sulfate contents of the polysaccharides. Kazłowski et al. (2012) conducted both in vitro and in vivo studies on Japanese encephalitis virus prevention property of novel SP from *Gracilaria* sp. and *M. nitidum*. During in vitro studies performed by MTT or plaque assays, low-degree-polymerization SP showed a remarkably high positive effect on survivability in JEV-infected C3H/HeN mice. The in vivo antiviral activity was assumed to be a resultant of better absorption of low-DP SP than undigested PS. The results support the feasibility of antiviral drug development from various SP and their derivatives.

### Antibacterial and antiprotozoan

Kumaran et al. (2010) studied that SP extracted from red alga *Kappaphycus alvarezii* and brown alga *Padina boergessenii* exert promising inhibitory response against antimicrobial-resistant *Escherichia coli* strains and, in particular, the inhibitory response of ampicillin-resistant *E. coli*, isolated from local fish markets and seafood processing plants. Maruyama et al. (2007) investigated the effects of fucoidan isolated from the sporophyll of *U. pinnatifida* on the *Cryptosporidium parvum* adhesion to the cultured human intestinal cells and its infection in neonatal mice. The *C. parvum* adhesion to human intestinal 407 cells was significantly suppressed by a low dose (1 mg/ml) of fucoidan (1 μg/ml). The results of the in vivo experiments revealed that *C. parvum* oocysts in the fucoidan-treated mice was reduced to nearly one-fifth of the oocysts number treated with phosphate buffered saline. It was concluded that fucoidan might inhibit cryptosporidiosis through the direct binding of fucoidan to the *C. parvum*-derived functional mediators in the intestinal epithelial cells in neonatal mice. Chen et al. (2009) investigated the inhibitory effects of fucoidan from the edible brown seaweed *U. pinnatifida*, on the growth of Plasmodium parasites. The antimalarial activity of fucoidan was assessed against the cultured *Plasmodium falciparum* parasites in vitro and on *Plasmodium berghei*-infected mice in vivo. Fucoidan significantly inhibited the invasion of erythrocytes by *P. falciparum* merozoites. Its 50 % inhibition concentration was similar to those for the chloroquine-sensitive *P. falciparum* 3D7 strain and the chloroquine-resistant K1 strain. Four-day suppressive testing in *P. berghei*-infected mice with fucoidan resulted in a 37 % suppressive effect versus the control group and a delay in death associated with anemia.

### Prevent hyperplasia

Hlawaty et al. (2011) investigated the therapeutic potential of low molecular weight fucoidan on vascular smooth muscle cell and human vascular endothelial cell proliferation and migration in vitro and in vivo. Sprague–Dawley rats with induced thoracic aorta injury were treated with SP (5 mg/kg/day) for 14 days. Results showed that SP prevented intimal hyperplasia in rat thoracic aorta. In situ zymography showed that the activity of matrix metalloproteinase (MMP)-2 in the neo-intima is significantly reduced. Fucoidans have been shown to mobilize bone marrow-derived progenitor cells via stimulation of stromal-derived factor (SDF)-1 release. Mobilized progenitor cells have been suggested to repair intimal lesions after immune-mediated endothelial injury and thus prevent intimal proliferation. Freguin-Bouilland et al. (2007) evaluated the therapeutic effect of these SP, in Brown Norway and Lewis rat aortic allograft model of transplant arteriosclerosis. The recipient rats were treated with SP (5 mg/kg/day) for 30 days. In contrast to untreated aortic allografts, the SP-treated allografts showed significantly less intimal proliferation. The SP treatment stimulated allograft reendothelialization, as evidenced by strong intimal endothelial nitric oxide synthase antibody and CD31 signals.

### Gastrointestinal functions

Graça et al. (2011) showed that a sulfated galactan isolated from red algae *Halymenia floresia* has promising effects on gastrointestinal (GI) motor functions mediated by voltage-gated Ca^2+^ channels. So, it is suggested that the SP can be useful when gastrointestinal contraction is necessary during motility-related disorders. Inflammatory bowel disease caused by enteric pathogens is a severe form of gastric disease characterized by excess production of proinflammatory cytokine IL-6. Fucoidan derived from brown algae *C. okamuranus* Tokida imparts LPS tolerance and prevents the expression of IL-6 mRNA as evidenced by in vitro and in vivo tests (Matsumoto et al. 2004).

### In regenerative and nano medicine

Sezer et al. (2008) prepared a fucoidan–chitosan hydrogel by swelling the polymers in acidic solution and investigated its dermal burn treatment efficiency. Dermal burns were inflicted on male New Zealand white rabbits and the prepared hydrogel was applied on the wounds. Histopathological evaluation of the biopsy samples was done at intervals. No edema was seen in tested groups after 3-day treatment and fibroplasia and scar were fixed after 7-day treatment. The best regeneration on dermal papillary formation and the fastest closure of the wounds were observed in fucoidan–chitosan hydrogels after 14-day treatment. Murakami et al. (2010) developed a hydrogel sheet by blending alginate, chitosan and fucoidan, for rapid wound healing. The hydrogel absorbed Dulbecco’s minimal essential medium (DMEM) and fluid absorption became constant within 18 h. On application, this hydrogel is expected to act as tissue adhesive and heal the wound in a moist milieu. Histological examination showed the advanced granulation tissue and capillary formation in the healing-impaired wounds treated with the hydrogel on day 7. Nakamura et al. (2008) reported that a chitosan/fucoidan complex-hydrogel enhanced the half life of fibroblast growth factor (FGF-2) by shielding it against denaturants as heat and proteolysis. Subcutaneous injection of the FGF-2-containing complex-hydrogel into the back of mice showed controlled release of bioactive protein. Slow diffusion of the growth factor induced neovascularization and fibrous tissue formation near the site of injection after 1 week. The complex-hydrogel was biodegraded after 4 weeks after supplying adequate amount of the angiogenic agents for protection of the ischemic heart. Fukuta and Nakamura (2008) reported that fucoidan and its oligosaccharides have the ability to stimulate production of hepatocyte growth factor (HGF) by induction during translation. So, it is believed that fucoidan may protect tissues and organs by mechanisms involving HGF.

Toskas et al. (2011) evaluated the nanofiber ability of an ulvan-rich extract from the alga *Ulva rigida*. Ulvan-based uniform, crystalline nanofibers of diameter 84 nm were produced by blending them with poly(vinyl alcohol) (PVA). The interesting biological and physicochemical properties of the nanofibers can lead to new biomedical applications such as drug release systems. Taken together, these findings indicate that the SP can revolutionize regenerative and nanomedicine, if exploited properly.

## Bottlenecks encountered

Extraction yield differs with respect to species, period and season of seaweed harvest (Robic et al. 2009). The SP are extracted from the seaweed biomass by many methods which influence their amount and chemical composition. The fucans of brown algae are highly complex and heterogeneous in structure, rendering their study difficult. Fonseca et al. (2008) compared the galactans from two species of red algae having same structure and size but slight variation in sulfation. Due to the variation in sulfate content, the two SP differed in their anticoagulant and venous antithrombotic activities. From the results it was concluded that slight differences in the proportions of sulfated residues in the galactan chain may be critical for the interaction between proteases, inhibitors and activators of the coagulation system. Also, the variations pose challenges in developing therapeutics. Furthermore, the high molecular weights of SPs pose issue in bio-availability (Jiao et al. 2011).

## Structure–function correlation of SP

It is important to understand the biochemical and molecular mechanism of therapeutic actions of SP, in order to develop effective drugs. The monomeric constituents, molecular size, sulfation site, specific structural motif, degree of branching determination are vital for reproducibility of result. Pomin (2009) has reported that the anticoagulant action of SP lies in its ability to inhibit plasma proteases via allosteric changes. The stereospecificities of the carbohydrate–protein complexes hinge on the number of residues in the repeating units, sulfation pattern, anomeric configuration, glycosidic linkage position and molecular mass. Also, the heterogeneities, such as acetylation, methylation and pyruvilation contribute in eliciting variations in functionality (Bilan et al. 2007). A single structural change has been traced to result considerable qualitative difference in results. Pomin and Mourao (2008) reported that preparation of oligosaccharides with well-defined chemical structures from sulfated fucan helps in the studies of carbohydrate–protein interaction. Fonseca et al. (2008) reported that algal sulfated galactans have a procoagulant effect along with the serpin-dependent anticoagulant activity. The procoagulant effect depends on the sulfation pattern of the SP. Slight differences in the proportions and/or distribution of sulfated residues along the galactan chain is critical for the interaction between proteases, inhibitors, and activators of the coagulation system, resulting in a distinct pattern in anti- and procoagulant activities. Identification of structural attributes of SP vital for their biological activities has been limited by their heterogeneous structures. Alasalvar et al. (2010) reported the strong correlation between structure of SP and their antioxidant potency. The monomeric constitution, degree of sulfation and their position, type of glycosidic linkage were held chief determining factors for variation in activity. High sulfate content and low molecular size were studied to exert stronger radical scavenging activities. Frenette and Weiss (2000) determined that sulfation is critical for efficacy of fucoidan in hematopoietic progenitor activity*.* The desulfated fucoidan failed to promote angiogenesis in vitro or to induce immature CD34+ cell mobilization in vivo. Fucoidan inhibits the human complement system mediated through interactions with certain proteins belonging to the classical pathway, particularly the protein C4. NMR spectra showed that the branched fucoidan oligosaccharides display a better anticomplementary activity compared to linear structures. Spectroscopy and molecular modeling of fucoidan oligosaccharides indicated that the presence of side chains reduces the flexibility of the backbone, mimicking a conformation recognized by the protein C4 (Clement et al. 2010). Leiro et al. (2007) observed that immunostimulatory activity of ulvan-like SP extracted from *U. rigida* was decreased significantly after desulfation of the SP, suggesting the importance of the functional group in eliciting immune response. To tackle the problem of heterogeneity of algal SP, a new approach has been established. The information obtained from studies of invertebrate SP that have a regular structure can be used to deduce the functionally of algal SP (Jiao et al. 2011).

## Maximization of the extraction and improvement in bioavailability

Aqueous (Ghosh et al. 2009) and acetone extraction (Marques et al. 2012) are the most prevalent techniques in SP production from seaweeds. Due to the variations in active growth parameters and extraction conditions, every new SP purified is a unique compound with signature structural features, promising a potential new drug. Rodriguez-Jasso et al. (2011) extracted fucoidan from brown seaweed *F. vesiculosus* by microwave-assisted extraction. Extraction at 120 psi, 1 min, using 1 g/25 ml water proved optimum condition for maximum fucoidan recovery. It was concluded that pressure, extraction time and alga/water ratio affected the SP yield (Rodriguez-Jasso et al. 2011). Supercritical CO_2_ extraction, ultrasonic-aid extraction and membrane separation technology may be applied to harvest SP from the seaweeds. Short extraction times, and non-corrosive solvents, cost effective an environmentally benign technique are required for maximum yield. Acid hydrolysis of high molecular weight fucans into low molecular weight compounds facilitates their structural investigation. Further, the low molecular weight fucoidans can be obtained by fucoidanase (E.C.3.2.1.44) treatment. This enzyme sourced from hepatopancreas of invertebrates, marine bacteria and fungi has an added advantage of hydrolyzing the SP without messing with its side substitute groups (Qianqian et al. 2011). Endolytic enzymes, such as ulvan lyases isolated from the flavobacteria *Persicivirga ulvanivorans* cleave the glycosidic bond between the sulfated rhamnose and a glucuronic or iduronic acid in the ulvans (Collen et al. 2011). Alkali modifications of carrageenans are suggested for improved application potential (Campo et al. 2009). Success of commercial reproducibility of highly diverse fucoidan lies in proper characterization with the help of powerful analytical tools (Fitton 2011).

## Conclusion

The research on SP from seaweeds and their wide biological spectrum have skyrocketed in recent years. Their clinical evaluation for possible noble therapeutics development is catching momentum like never before. For above goals to materialize, the underlying molecular mechanisms need to be understood precisely and elucidated clearly. The relation between structure and function should be unraveled by intensive studies. This up-to-date review on this emerging technique is expected to contribute significantly in supplementing background knowledge, kindling interest for future explorations. Further purification steps and investigation on structural features as well as in vivo experiments are needed to test the viability of their use as therapeutic agents. The SP with appreciably few side effects and myriad benefits could potentially be exploited for complementary medicine use and disease management.
